# „I ride with you“ – active promotion of mental health in Bulgaria

**DOI:** 10.1192/j.eurpsy.2022.1785

**Published:** 2022-09-01

**Authors:** K. Guevara

**Affiliations:** State Psychiatric Hospital for treatment of drug addiction and alcoholism, Acute Ward, Sofia, Bulgaria

**Keywords:** cause, sport, Bulgaria, mental health promotion

## Abstract

**Introduction:**

One of the main concepts of mental health promotion is proactivity, rather than reactivity. As psychiatrists we must be the first line of mental health advocacy and to do so we must share clear messages and take definitive actions.

**Objectives:**

Inventing a cause that raises mental health awareness in the society. Afterwards creating a page in the social media that uses common language and which represents the main concepts of mental health and also targets prevention and treatment of mental disorders. Taking part in sport events that have the potential to increase the social awareness and reaching national media in order to popularize the cause.

**Methods:**

First came creation of a page in the social media. Afterwards came the preparation of video materials and e-posters on the following topics: mental health, stigma, myths and facts about mental disorders, early trauma, mental disorders. The materials were posted and “boosted”. All this was accompanied by numerous media events and interviews on national media. In order to garner more attention there were two participations in a 750km bike ultra marathons.

**Results:**

The complex approach of the cause „I ride with you” (Az Karam s teb) led to the establishment of a popular page in the social media. Within 1 year the page got 1500 followers. The page content was shared 733 times. There were 12 national media appearances. All these numbers represent a small but significant step in mental health promotion.

**Conclusions:**

In order to promote healthy “mental environment” we must use diverse and contemporary approaches.

**Disclosure:**

No significant relationships.

